# Resilience to Chronic Stress Is Characterized by Circadian Brain-Liver Coordination

**DOI:** 10.1016/j.bpsgos.2024.100385

**Published:** 2024-08-23

**Authors:** Christina Savva, Ivan Vlassakev, Blynn G. Bunney, William E. Bunney, Lucas Massier, Marcus Seldin, Paolo Sassone-Corsi, Paul Petrus, Shogo Sato

**Affiliations:** a Department of Medicine (H7), Karolinska Institutet, Stockholm, Sweden; b Department of Psychiatry and Human Behavior, School of Medicine, University of California, Irvine, Irvine, California; c Helmholtz Institute for Metabolic, Obesity and Vascular Research (HI-MAG) of the Helmholtz Zentrum München at the University of Leipzig and University Hospital Leipzig, Leipzig, Germany; d Department of Biological Chemistry, School of Medicine, University of California, Irvine, Irvine, California; e Center for Biological Clocks Research, Department of Biology, Texas A&M University, College Station, Texas

**Keywords:** Chronic social stress, Circadian rhythms, Gene expression, Metabolism, Mouse model for depressive-like behaviors, Multitissue omics analysis

## Abstract

**Background:**

Chronic stress has a profound impact on circadian regulation of physiology. In turn, disruption of circadian rhythms increases the risk of developing both psychiatric and metabolic disorders. To explore the role of chronic stress in modulating the links between neural and metabolic rhythms, we characterized the circadian transcriptional regulation across different brain regions and the liver as well as serum metabolomics in mice exposed to chronic social defeat stress, a validated model for studying depressive-like behaviors.

**Methods:**

Male C57BL/6J mice underwent chronic social defeat stress, and subsequent social interaction screening identified distinct behavioral phenotypes associated with stress resilience and susceptibility. Stressed mice and their control littermates were sacrificed every 4 hours over the circadian cycle for comprehensive analyses of the circadian transcriptome in the hypothalamus, hippocampus, prefrontal cortex, and liver together with assessments of the circadian circulatory metabolome.

**Results:**

Our data demonstrate that stress adaptation was characterized by reprogramming of the brain as well as the hepatic circadian transcriptome. Stress resiliency was associated with an increase in cyclic transcription in the hypothalamus, hippocampus, and liver. Furthermore, cross-tissue analyses revealed that resilient mice had enhanced transcriptional coordination of circadian pathways between the brain and liver. Conversely, susceptibility to social stress resulted in a loss of cross-tissue coordination. Circadian serum metabolomic profiles corroborated the transcriptome data, highlighting that stress-resilient mice gained circadian rhythmicity of circulating metabolites, including bile acids and sphingomyelins.

**Conclusions:**

This study reveals that resilience to stress is characterized by enhanced metabolic rhythms and circadian brain-liver transcriptional coordination.

## Plain Language Summary

Chronic stress can have detrimental effects on both physical and mental health, often disrupting biological daily rhythms, known as circadian rhythms. To delve deeper into this phenomenon, we investigated how chronic stress affects circadian rhythms in the brain, liver, and blood metabolism of mice. Our study revealed that mice resilient to stress showed an increase in shared circadian biological processes between the liver and different brain regions together with enhanced rhythms in circulating metabolites. These findings propose an unprecedented link between stress adaptation and systemic circadian coordination and offer valuable insights into the mechanisms that underlie circadian disturbances seen in psychiatric disorders.

The increasing prevalence of depressive-like symptoms and the role of depression as a strong risk factor for suicide make it a challenging public health problem. Therefore, understanding the molecular mechanisms that underlie depression could lead to the identification of novel targets for therapeutic interventions. Chronic stress contributes to the development of various psychiatric symptoms and can be used as a model to dissect the mechanisms that contribute to depression (1). Furthermore, chronic stress influences the circadian system, which is believed to be an important etiological factor in depression (2,3). Epidemiological and molecular evidence points to the interaction between psychiatric disorders and circadian rhythm disruptions (4, 5, 6, 7). Moreover, circadian disruption also contributes to metabolic diseases (8), and emerging evidence points toward shared mechanisms contributing to metabolic and depression comorbidity (9). Therefore, parallel exploration of the circadian-metabolic and circadian-neuronal response to stress-induced depression may elucidate novel intervention strategies for depression.

Circadian rhythms govern the temporal dynamics of multiple physiological and behavioral functions (10). The circadian system operates autonomically at the single-cell level, originating from the genetically encoded core clock machinery (11). Light is the main environmental cue that entrains the circadian clock (zeitgeber, from the German "time-giver") and thus synchronizes the clock in the hypothalamic suprachiasmatic nucleus (12). The suprachiasmatic nucleus serves as a central pacemaker that orchestrates circadian rhythms by communicating the photic cues to other brain regions and peripheral clocks throughout the body. These tissue-specific clocks collectively form a federated system that facilitates temporal communication, thereby regulating circadian physiology (13). Thus, circadian disruption of neural rhythms could influence peripheral metabolic oscillations and vice versa (8,14,15). These intricate interactions suggest the presence of potential circadian mechanisms responsible for brain-body communication (16).

In animal models, chronic social defeat stress (CSDS) is a standard paradigm used to study the molecular mechanisms that underlie depressive-like behaviors and antidepressant actions in a translationally relevant manner (17,18). Mice undergoing CSDS exhibit stress-resilient and stress-susceptible phenotypes, mimicking diverse responses to stress in humans and allowing for in-depth molecular investigations into these distinct phenotypes. An important outstanding question remains: Why does chronic stress adversely affect mood in some patients, while other individuals are resilient (19)? In this study, we utilized the CSDS model and a multi-omics approach to explore how circadian rhythms respond to CSDS. Our data suggest that resilience to stress is accompanied by gained transcriptional rhythmicity in both the hypothalamus (HT) and the liver. Furthermore, stress-resilient mice exhibited robust pathway coordination of circadian transcripts between these 2 tissues. These observations were corroborated by gained circulatory metabolic rhythms. We propose that circadian circulatory metabolites can serve as signaling molecules to mediate stress resilience and represent potential novel targets for treatment of psychiatric disorders.

## Methods and Materials

### Animals

Male 8-week-old C57BL/6J mice and 6-month-old CD-1 mice that were retired breeders were purchased from Jackson Laboratories and Charles River Laboratories, respectively. Mice were fed a standard rodent laboratory diet (2020X Teklad Global Extracted Rodent Diet) ad libitum and reared at 24 to 25 °C on a 12-hour light/dark cycle. Animals were maintained at the animal facilities of the University of California, Irvine. Animal experiments were approved by the Institutional Animal Care and Use Committee of the University of California, Irvine (AUP-18-022).

### Chronic Social Defeat Stress

**Figure 1:**
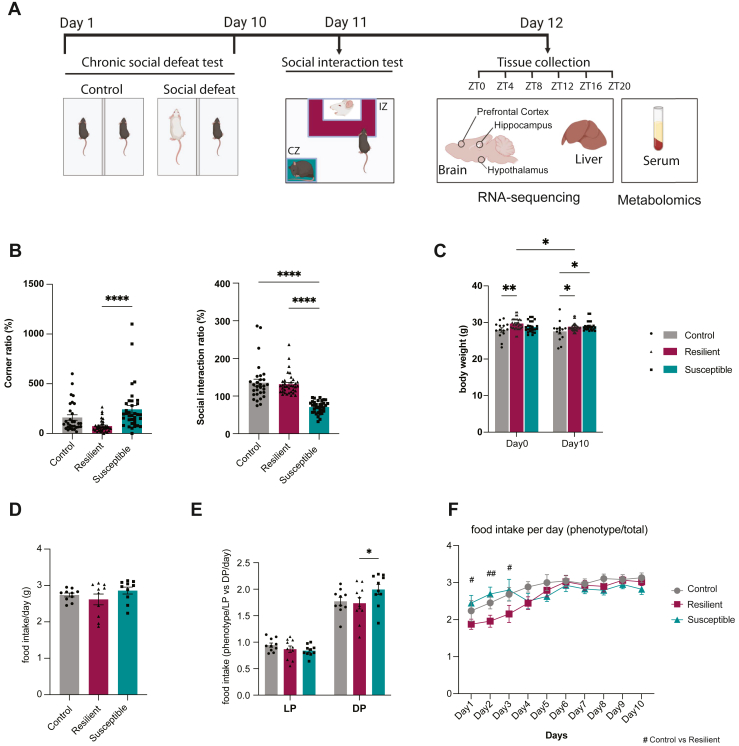
Phenotyping of mice following chronic social defeat stress. **(A)** Schematic overview of the experimental design and the timeline. Days 1–10, chronic social defeat test; day 11, social interaction test; and day 12, sacrifice and tissue collection over the circadian cycle for RNA sequencing and metabolomics. **(B)** Social interaction test score showing the ratio in time spent in the CZ and IZ in the presence and absence of the aggressor. *n* = 30 in the control group, and *n* = 36 in the resilient and susceptible groups. **(C)** Body weight (g) on day 0 and day 10. *n* = 14 controls, *n* = 33 resilient, and *n* = 23 susceptible. **(D)** Average food intake (g) per day. *n* = 14 controls, *n* = 17 resilient, and *n* = 13 susceptible. **(E)** Average food intake (g) during light and dark periods. *n* = 10 per group. **(F)** A 10-day time course of food intake (g). *n* = 14 controls, *n* = 17 resilient, and *n* = 13 susceptible. Two-way analysis of variance followed by Tukey’s post hoc test. Data are presented as mean ± SEM. ∗*p* < .05, ∗∗*p* < .01, and ∗∗∗∗*p* < .0001. For **(F)**, *#p* < .05 and ##*p* < .01 for the resilient vs. control group comparison. Control (gray), resilient (purple), and susceptible (blue) mice. CZ, corner zone; DP, dark period; IZ, interaction zone; LP, light period; ZT, zeitgeber time.

An established CSDS protocol was used to induce depressive-like behaviors in mice (20, 21, 22) (Figure 1A).

#### Screening of Aggressor

The screener C57BL/6J mice were placed directly into the home cage of the aggressor CD-1 mouse for 180 seconds with the aggressor present. We performed 3 screening sessions at zeitgeber time (ZT) 0, corresponding to the onset of light, once daily, using different screeners on each subsequent day for each aggressor such that no aggressor defeated the same screener twice.

#### Social Defeat Protocol

A total of 90 male 2- to 3-month-old C57BL/6J mice were included in the study. The mice were housed in a shared home cage with the larger aggressive CD-1 mouse for the duration of the experiment. The home cage had a clear perforated divider so that the smaller mice were continuously exposed to the CD-1 mouse. For the social defeat protocol, the C57BL/6J mouse (intruder) was placed directly in the compartment with the CD-1 mouse (resident aggressor) for 10 minutes on 10 consecutive days (Figure 1A). The social defeat exposure was conducted at ZT0. After 10 minutes of social defeat, the C57BL/6J mouse was transferred across the perforated divider to the opposite compartment and housed in this compartment for 24 hours. For each subsequent daily social defeat, the C57BL/6J mouse was exposed to a novel resident’s home cage compartment to prevent any habituation to the residential aggressor. Control C57BL/6J mice were placed in an identical home cage setup; a pair of control mice per side were divided by a cage divider for the duration of the defeat sessions. The control mice were rotated to a new cage daily. Food intake and body weight were measured daily throughout the CSDS period.

#### Social Interaction Test

After 10 days of CSDS, we carried out a social interaction test (SIT) to evaluate whether mice exhibited susceptible or resilient phenotypes. Each SIT was composed of two 150-second phases, separated by a duration of 30 seconds, either with or without the target aggressive CD1 mouse present in the interaction zone. We measured the time spent in the interaction zone during the first (target absent) and second (target present) trials; the interaction ratio was then calculated as 100 × (interaction time, target present)/(interaction time, target absent). The SIT was performed at ZT0.

#### Circadian Tissue Harvest

After 24 to 44 hours of SIT, the whole-brain including the prefrontal cortex (PC), hippocampus (HC), and HT together with the liver were collected every 4 hours throughout the circadian cycle (ZT 0, 4, 8, 12, 16, and 20). Serum samples were also collected throughout the circadian cycle from the mice undergoing CSDS. The collected tissues were immediately frozen in liquid nitrogen.

### RNA Isolation, Library Preparation, and RNA Sequencing

Total RNA was extracted from brain and liver tissues using TRIzol (Invitrogen). Next, RNA samples were tested for concentration and quality, and samples where the RNA integrity number was >7.0 were used in downstream applications. Libraries were prepared using NEBNext Ultra RNA Library Prep kits per the manufacturer’s instructions. A total of 800 to 1000 ng of RNA was used for library preparation with 12 polymerase chain reaction cycles. The resultant libraries were tested for quality via bioanalyzer. Individual libraries were pooled and sequenced using Novogene 6000 (Novogene). Raw RNA sequencing (RNA-seq) reads were inspected for quality using FastQC version 0.11.9 (Barbraham Institute). Reads were aligned to the current mouse genome (GRCm38) using standard STAR aligner settings, and polymerase chain reaction duplicates were removed using MarkDuplicates (Picard Tools). Counts were then assembled from BAM files used to summarize overlaps in function at the gene level from mouse transcriptome GFF file and also from (GRCm38). Raw RNA-seq data has been deposited into Mendeley Data (https://data.mendeley.com/datasets/fx29crw2fv/2).

### Metabolomics

Metabolomic analysis was performed by Metabolon, Inc. Briefly, samples were prepared using the automated MicroLab STAR system. All methods utilized Waters ACQUITY ultraperformance liquid chromatography and a Q-Exactive high resolution/accurate mass spectrometer (Thermo Fisher Scientific) interfaced with a heated electrospray ionization source and Orbitrap mass analyzer operated at 35,000 mass resolution. Raw data was extracted, the peak identified, and quality control processed using Metabolon’s proprietary hardware and software (23,24). Compounds were identified by comparison to library entries of purified standards or recurrent unknown entities. Raw metabolomics data has been deposited into Mendeley Data (https://data.mendeley.com/datasets/fx29crw2fv/1).

### Data Analyses and Statistics

#### Rhythmicity Analysis

The *DryR* package (25) was used to identify differential rhythmicity in RNA-seq data collected in a time series using raw count data for each tissue, with default parameters. *DryR* function implements a rhythmicity analysis based on a generalized linear model with a subsequent model selection using the Bayesian information criterion. Metabolomic analysis of serum samples collected in the same time series as the tissues were sent to Metabolon. The log-transformed data from Metabolon was used to identify rhythmic metabolites with the *DryR* package, using the *drylm* function. Outliers were eliminated based on a principal component analysis.

#### Pathway Annotation Analysis of Differentially Rhythmic Genes

To identify biological processes and Kyoto Encyclopedia of Genes and Genomes pathways of the differentially rhythmic genes in the collected tissues, we used the ClusterProfiler (26) package. Genes belonging to models 2, 3, 5, and 9 generated by *DryR* were used to generate pathway analysis with a cutoff of *p* value < .05 and a Benjamini-Hochberg–adjusted *p* value < .05. The overlap between the genes in each tissue, presented by Circos plots, were created by submitting gene lists generated by *DryR* to the online tool Metascape (27).

#### Cell Type–Specific Analysis

Cell type–specific transcripts were extracted from a previously published hypothalamic single-cell RNA-seq dataset (28). These data were used to extract the cell-specific circadian transcriptome in the HT of the control, resilient, and susceptible mice.

#### Statistics

The statistical comparisons were performed using GraphPad Prism version 10.0.0, unless otherwise stated. Data were analyzed using either ordinary 1-way or 2-way analysis of variance followed by Tukey’s multiple comparison tests, with the significance threshold set at *p* < .05. The data are presented as mean ± SEM.

## Results

Based on the results of the SIT (Figure 1A, B), mice were subdivided into 2 groups: those exhibiting resilience to stress and those showing susceptibility to stress. No differences in body weight were observed between resilient and susceptible mice. However, both groups had higher body weight than the controls on day 10 (Figure 1C), although no significant differences in total food consumption were observed (Figure 1D). Increased consumption of food in the susceptible group occurred during the dark period (Figure 1E), when mice were active, and manifested during the 3 to 5 days of the intervention (Figure 1F). These results support the idea of a causal link between mood and metabolism.

### CSDS Reprograms the Circadian Transcriptome of the Brain

**Figure 2:**
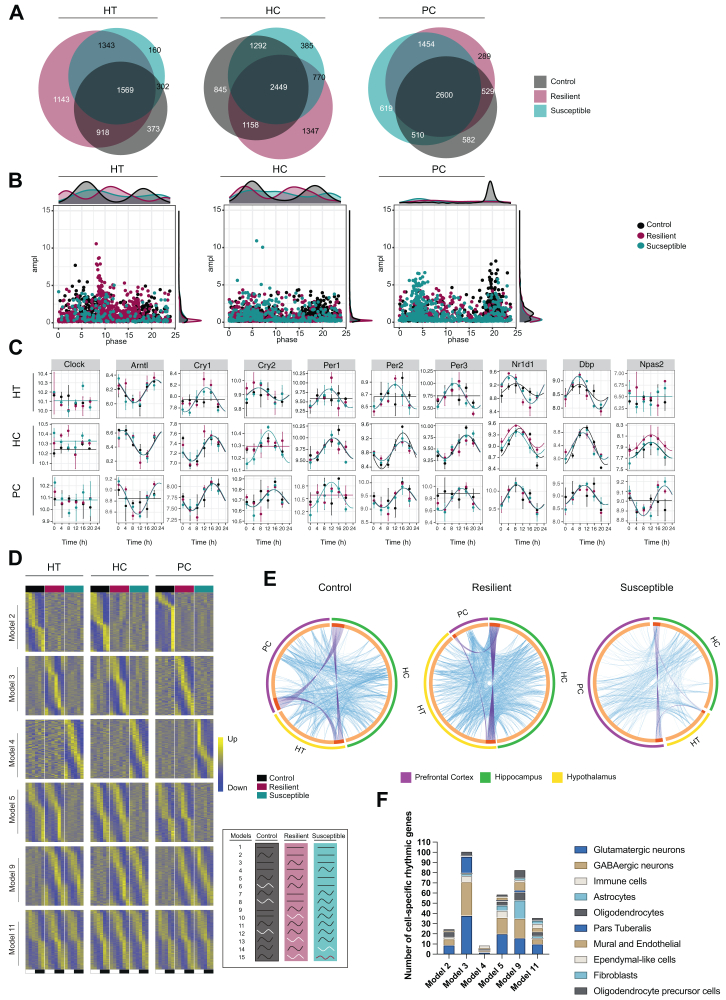
The circadian transcriptome of the brain is rewired by chronic social defeat stress. **(A)** Venn diagrams showing the overlap of rhythmic genes between control, resilient, and susceptible mice in the HT, HC, and PC. **(B)** Scatter plots representing the distribution of exclusively rhythmic genes from each group in the 3 brain regions together with the respective marginal histograms. The y-axis and x-axis represent the amplitude and phase, respectively. **(C)** Rhythmic expression of core clock genes in the brain. The y-axis represents the log_2_-normalized counts, and the x-axis represents the time (zeitgeber time). Fitted lines represent significant rhythmicity, while flat lines represent non-rhythmicity. **(D)** Heatmaps of normalized rhythmic messenger RNA levels in the 3 brain regions. The box to the right of the heatmaps represent all models in the DryR analyses outputs. Model 1, nonrhythmic in all; model 2, rhythmic only in control; model 3, rhythmic only in resilient; model 4, rhythmic only in susceptible; models 5 and 6, rhythmic in control and resilient but not in susceptible; models 7 and 8, rhythmic in control and susceptible but not in resilient; models 9 and 10, rhythmic in resilient and susceptible but not in control; models 11–15, rhythmic in all. Higher and lower amplitudes are indicated by yellow and blue color, respectively. Flat lines represent no rhythms, and black waves represent significant circadian rhythmicity. White waves represent antiphasic rhythms in relation to the other group with rhythmic transcripts within the same model. Model 15 represents different rhythmic patterns between all 3 groups, hence the inclusion of a red wave line. **(E)** Circos plots showing the overlap and interconnection of rhythmic genes from each brain region in control, resilient, and susceptible mice. The outer layer of the circle represents the gene list of PC (purple), HC (green), and HT (yellow). The dark orange in the inner side of the circle represents the genes that are shared, and the light orange color shows the genes that are unique to certain brain regions. The purple lines connecting the genes show the same genes shared between the gene lists, and the blue lines link the genes that belong to the same ontology. **(F)** Stacked bar chart representing the cell type–specific hypothalamic rhythmic transcripts within each model. The cell-type specificity was extracted from previously published single-cell RNA sequencing data of mouse hypothalamus. *n* = 3 mice per time point and group. GABAergic, gamma-aminobutyric acidergic; HC, hippocampus; HT, hypothalamus; PC, prefrontal cortex.

To rigorously dissect tissue-specific circadian transcriptomic responses to CSDS, we carried out a comprehensive analysis of circadian gene expression in 3 brain regions relevant to depression: the HT, HC, and PC, isolated throughout the circadian cycle. All 3 brain regions showed a large proportion of circadian transcripts with a profound overlap of shared oscillating genes between the groups in the HT (1569 genes), HC (2449 genes), and PC (2600 genes) (Figure 2A and Supplement 2). Importantly, chronic stress induced a robust circadian reprogramming in a brain region–specific manner: stress-resilient mice exhibited a greater number of de novo cycling genes in the HT and HC, whereas susceptible mice gained oscillating transcripts in the PC, although to a smaller extent. Moreover, the phase and amplitude of rhythmic transcripts varied between the groups and across different brain regions (Figure 2B). Susceptible mice displayed a range of gene expression peaks over the circadian cycle in the HT and HC, while their PC had a distinct peak in gene expression around ZT4. Conversely, control and resilient mice exhibited distinct peaks in the HT and HC, but with phase shifts between these groups. Circadian clock genes were profiled to assess the impact of CSDS on the core clock machinery in the brain (Figure 2C). The expression of multiple clock genes did not exhibit circadian oscillation in the brain, which is consistent with a previous report (29). In fact, many clocks did not display rhythmicity in control mice but gained significant circadian rhythms in mice exposed to the aggressors. These included genes in the HT (*Cry1*, *Per1-3*) and PC (*Arntl*, *Per3*, and *Npas2*). In addition, *Cry2* in the HC and *Per1* in the PC were the only clock genes that were differentially rhythmic in susceptible versus resilient mouse brains. Taken together, these data suggest that exposure to stress alters the rhythmicity of specific genes within the core clock machinery in the brain mainly by inducing circadian rhythms in stressed mice, with only minor differences between susceptible and resilient mice.

To further dissect the circadian transcriptional response to CSDS, transcripts were analyzed using the DryR algorithm that provides an output with models categorizing the transcripts based on their rhythmic expression patterns (Figure 2D). Comparison between the control, resilient, and susceptible rhythmic transcriptomes provided up to 15 different models (the box to the right of the heatmaps in Figure 2D describes the rhythmic patterns of each group in the different models). We focused on models 2 to 5, 9, and 11 because these were considered the best-fitted models to distinguish between the effects of chronic stress and the circadian output to stress associated with the susceptible/resilient phenotype. Pathway analysis of model 2 (genes cycling only in control mice) highlighted the PC and HC genes involved in generation of precursor metabolites and energy and the HT genes involved in response to nutrient levels that lost their rhythmicity in response to social stress (Figure S1 in Supplement 1). Furthermore, stress-resilient brains (model 3) gained rhythmicity in genes involved in synapse function in the HC and HT (Figure S2 in Supplement 1), and similar pathways were found in pathway enrichment analyses performed on the genes oscillating in both control and resilient brains (model 5) (Figure S3 in Supplement 1). In the model with rhythmic genes induced by stress (shared genes in resilient and susceptible mice, model 9), the PC and HC showed an enrichment of actin filament rhythms, while the HT involved lipid localization (Figure S4 in Supplement 1). These data provide information on the region-specific response to stress; however, the neuronal response to stress is regulated by coordination across brain regions (30). To explore the temporal transcriptional coordination across brain regions, we assessed the proportion of oscillating genes annotated in shared pathways. Remarkably, we observed a substantial increase in temporal transcriptional coordination in stress-resilient brains compared with brains in the other groups (Figure 2E). Notably, resilient mice showed pronounced connections to other brain regions originating from the HT. The enhanced transcriptional rhythmicity and interconnection with other brain regions of the HT in the resilient group led us to investigate the cell specificity of these oscillations. To this end, we overlapped the rhythmic genes in the different models with a previously published single-cell RNA-seq dataset (28). Both GABAergic- (gamma-aminobutyric acidergic) and glutamatergic-specific genes were oscillating in all models except model 4 (genes rhythmic only in susceptible HT), which in general displayed few rhythmic cell-specific markers (Figure 2F). Conversely, model 3 (rhythmic specifically in resilient HT) uniquely involved an enrichment for rhythmic genes expressed in cells specific to the pars tuberalis of the pituitary gland. In addition, the models that included rhythmic genes shared by the resilient and either control or susceptible HT (models 5 and 9) were enriched for astrocyte markers. These findings collectively suggest that hypothalamic rhythmic gene regulation may contribute to the circadian adaptation to social stress through metabolic and/or neuroendocrine signaling.

### Resilience to Social Defeat Is Characterized by De Novo Hepatic Transcriptional Rhythms

**Figure 3:**
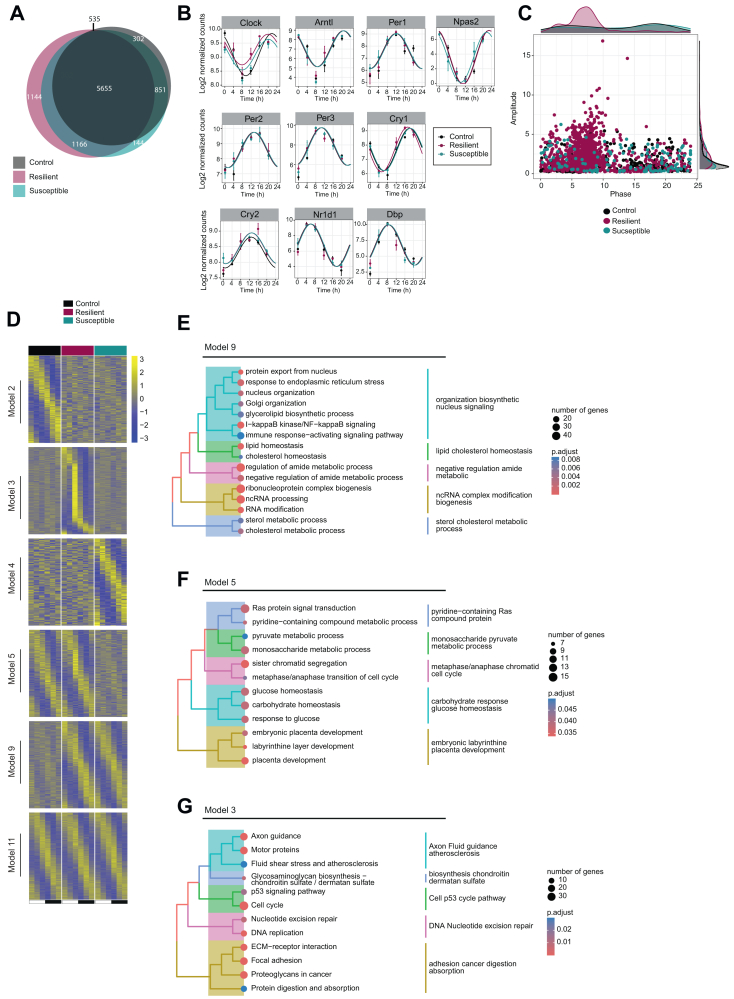
Social stress impacts the liver circadian transcriptome. **(A)** Venn diagram showing the overlap of rhythmic genes between control, resilient, and susceptible mice in the liver. **(B)** Rhythmic expression of core clock genes in the liver. The y-axis represents the log_2_-normalized counts, and the x-axis represents the time. **(C)** Scatter plot representing the distribution of exclusively rhythmic genes from each group in the liver together with the respective marginal histograms. The y-axis represents the amplitude, and the x-axis represents the phase. **(D)** Heatmaps of normalized rhythmic messenger RNA levels in the liver in control, resilient, and susceptible groups from model 11, rhythmic in all; model 2, rhythmic only in control; model 3, rhythmic only in resilient; model 4, rhythmic only in susceptible; model 5, rhythmic in control and resilient but not in susceptible; and model 9, rhythmic in resilient and susceptible but not in control. Higher and lower amplitudes are indicated by yellow and blue, respectively. Explanation for the models is visualized in Figure 2D. **(E–G)** Tree plot presenting a hierarchical clustering of enriched terms from input gene lists from model 9 [**(E)** resilient- and susceptible specific], 5 [**(F)** control- and resilient specific], and model 3 [**(G)** resilient specific] together with the expression patterns of selected genes belonging to chosen pathways from each model. The background colors indicate a different hierarchical group of enriched pathways. The bubble size shows the enrichment size, and the color of the bubble shows the significance (adjusted *p* value cutoff < .05). *n* = 3 mice per time point and group. ECM, extracellular matrix; ncRNA, noncoding RNA; NF, nuclear factor.

The HT plays a vital role as a central regulator of feeding/fasting cycles, orchestrating temporal appetite control (31). Furthermore, the HT houses the suprachiasmatic nucleus, which coordinates circadian functional activities in peripheral tissues such as liver (32). Circadian regulation plays a significant role in hepatic metabolism because essential metabolic pathways, including those responsible for carbohydrates, lipids, cholesterol, and bile acids, exhibit daily oscillations in the liver. Given the intriguing association between stress resilience and hypothalamic transcriptional rhythms (Figure 2A, E), we embarked on an investigation into the impact of chronic social stress on the liver circadian transcriptome. Consistent with the HT transcriptome, resilient mice showed dramatic de novo hepatic transcriptional oscillations (1144 genes) (Figure 3A and Supplement 2) without influencing the rhythmicity of the core clock genes (Figure 3B). The gained oscillations in the liver of resilient mice displayed a distinct peak at ZT8 (Figure 3C), which again is in agreement with the observations made in the HT. The same categorization models as described for the brain were employed for the liver (Figure 3D) and subsequently conducted pathway analyses. Genes oscillating specifically in control livers (model 2) did not yield any significantly enriched pathways. Conversely, the liver from stressed mice (model 9) gained rhythmicity in several pathways, including cholesterol metabolism (Figure 3E). In contrast, susceptible mice displayed a loss of daily oscillation in genes involved in glucose and pyruvate metabolism in the liver (model 5) (Figure 3F). Resilient-specific rhythms were not enriched for any metabolic pathways but instead exhibited enrichment in pathways such as axon guidance and cell cycle (Figure 3G). Taken together, these findings provide a comprehensive overview of the hepatic circadian response to stress and suggest that enhanced transcriptional rhythms characterize resilience to stress.

**Figure 4:**
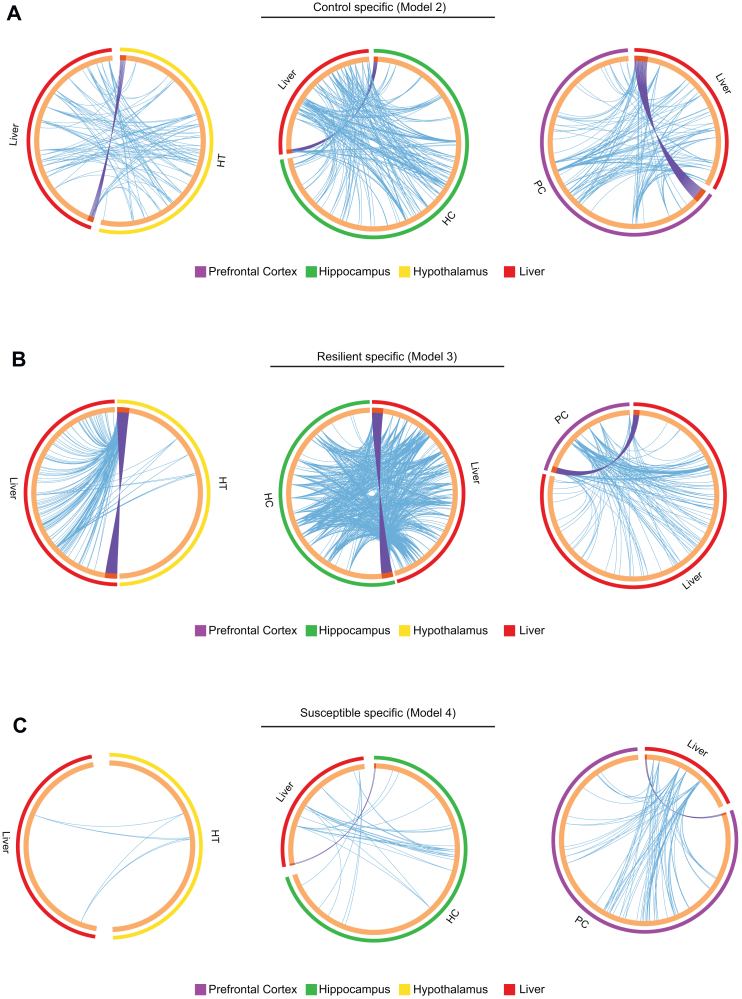
Brain-liver coordination of circadian transcriptional pathways. **(A–C)** Circos plots representing the overlap and interconnection of rhythmic genes between the liver and the different brain regions in **(A)** model 2, rhythmic only in control; **(B)** model 3, rhythmic only in resilient; and **(C)** model 4, rhythmic only in susceptible. The outer layer of the circle represents the gene list from each tissue: liver (red), PC (purple), HC (green), and HT (yellow). The dark orange in the inner side of the circle represents the genes that are shared between the tissues, and the light orange color shows the genes that are unique in each tissue. The purple lines connecting the genes show identical genes shared between the tissues, and the blue lines link the genes that belong to the same ontology. *n* = 3 mice per time point and group. HC, hippocampus; HT, hypothalamus; PC, prefrontal cortex.

Circadian coordination between brain and peripheral tissues plays a pivotal role in orchestrating organismal circadian rhythmicity and maintaining physiological homeostasis (33). To explore the temporal coordination between the liver and the brain, we investigated the shared pathways within the rhythmic transcriptome between the liver and different brain regions. Resilient mice exhibited the most pronounced connectivity between the liver and the HT (Figure 4A–C, left panels). Furthermore, in both control and resilient mice, the hippocampal circadian transcriptomes shared numerous pathways with those of the liver (Figure 4A–C, middle panels). These findings are consistent with the observations presented in Figures S1 to S4 in Supplement 1, which suggest that metabolic pathways exhibited rhythmicity in the HC. In stark contrast, susceptible mice had few hepatic genes that shared pathways with the brain (Figure 4C). Collectively, our results highlight the profound effect of social defeat on circadian gene regulation in the liver.

### Resilience to Social Defeat Is Associated With Rhythmicity of Circulating Bile Acids

**Figure 5:**
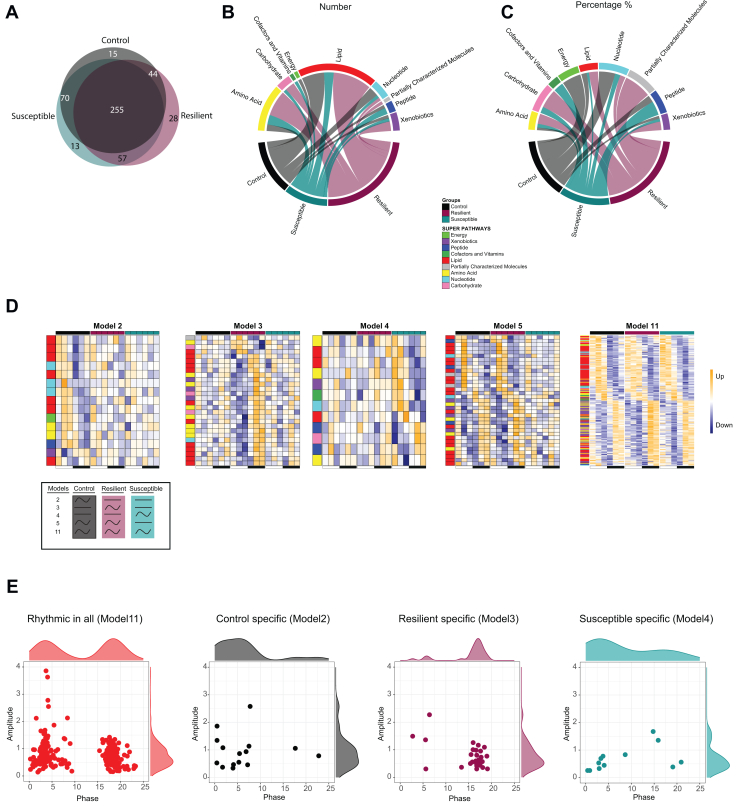
Resilience to social stress is characterized by enhanced rhythmicity of circulatory metabolites. **(A)** Venn diagram showing the overlap of rhythmic metabolites between the control, resilient, and susceptible mice in the serum. **(B, C)** Chord plot showing the **(B)** number and **(C)** percentage of rhythmic metabolites belonging to super pathways in control, resilient, and susceptible groups. **(D)** Heatmaps of log-normalized metabolite abundancy in the serum of the control, resilient, and susceptible mice from model 11, rhythmic in all; model 2, rhythmic only in control; model 3, rhythmic only in resilient; model 4, rhythmic only in susceptible; and model 5, rhythmic in control and resilient but not in susceptible. Explanation for the models is visualized in Figure 2D. Higher and lower amplitudes are indicated by orange and blue, respectively. Row annotations show the super pathways of each metabolite. **(E)** Scatter plots representing the distribution of metabolites from model 11 (rhythmic in all), model 2 (control specific), model 3 (resilient specific), and model 4 (susceptible specific) with the respective marginal histograms. The y-axis represents the amplitude, and the x-axis represents the phase. *n* = 4–5 mice per time point and group.

**Figure 6:**
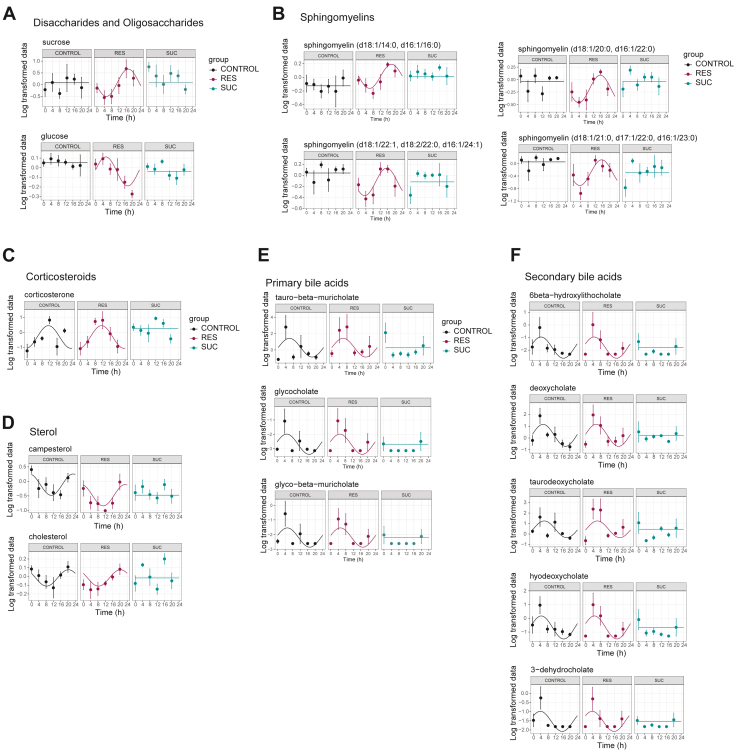
Circadian metabolic pathways in response to chronic social defeat stress. **(A–F)** Relative levels of metabolites in **(A)** disaccharide and oligosaccharide metabolism, **(B)** sphingomyelin metabolism, **(C)** corticosterone metabolism, **(D)** sterol metabolism, **(E)** primary bile acids, and **(F)** secondary bile acids in the serum collected from the control, RES, and SUS mice. The y-axis represents the log-transformed values, and the x-axis represents the time. *n* = 4–5 mice per time point and group. RES, resilient; SUC, susceptible.

Circadian inter-organ coordination underlies the maintenance of systemic metabolic homeostasis (13,34,35). In particular, the HT plays an important role in regulating systemic metabolism, in part by communicating with the liver via both endocrine signaling and the peripheral nervous system (36). Therefore, we reasoned that the gain of transcriptional rhythms in the liver and hypothalamus would be reflected by gained rhythmicity of systemic metabolism. Therefore, next we explored circulating metabolite rhythms by performing untargeted metabolomics analyses. Consistent with the liver-hypothalamus transcriptional coordination, resilient mice exhibited the highest proportion of de novo oscillating metabolites (Figure 5A and Supplement 2). Resilient mice had a large proportion of rhythmic levels of lipids, carbohydrates, and amino acids, while susceptible mice had a larger proportion of oscillating vitamins and peptides (Figures 5B, C). The circadian circulating metabolome displayed 2 distinct peaks around ZT4 and ZT16 (Figures 5D, 5E), consistent with previous findings (37). Interestingly, most of the metabolites that showed unique oscillations in control and susceptible mice peaked around ZT4, while those unique to resilient mice reached their peak at around ZT16. Among the metabolites exclusively oscillating in resilient mice, sucrose and glucose exhibited antiphase oscillation with each other, with glucose peaking at ZT4 and sucrose peaking at ZT16 (Figure 6A). Another prominent group of oscillating metabolites in resilient mice consisted of sphingomyelins (Figure 6B). Metabolites that lost oscillation in the serum in the susceptible mice included steroid metabolite classes (Figure 6C–F). Transcriptional rhythmicity of cholesterol metabolism in the liver was induced by stress exposure (Figure 3E), suggesting that the absence of the temporal dynamics of steroid metabolites is independent of the circadian regulation of gene expression involved in cholesterol metabolism and that other organs, such as the gut, are involved in regulating the circulatory rhythms of the metabolites. This loss of rhythmicity encompassed both primary and secondary bile acids, as well as sterols and corticosterone, all of which are signaling molecules potentially implicated in mediating neuroprotective functions (38). These molecules could serve as liver-brain signaling molecules essential for coping with the stress induced by social defeat. In summary, our comprehensive circadian transcriptome and metabolome in socially defeated mice revealed systemic circadian metabolic consequences.

## Discussion

The brain continuously receives external environmental cues to fine-tune physiology and behaviors. Chronic stress alters the transcriptional response in several brain regions (22,39) and affects physiological rhythms (40,41). Furthermore, brain-body communication operates under circadian control (42), and it is therefore important to explore the temporal aspects of the stress response. In this study, we explored the temporal aspects of the transcriptional response as well as the systemic metabolic response to chronic stress. Our results revealed that CSDS had profound effects on the circadian transcriptome of the brain and the liver. Importantly, we demonstrated that resilience to stress was associated with a gain in the circadian rhythmicity of the hypothalamic and hepatic transcriptomes. The coordination of transcriptional pathways between the HT and liver could be controlled via multiple routes and involve neuroendocrine signaling as well as neuronal signaling via the peripheral nervous system (16,36).

Exposure to stress engages various brain regions that collectively orchestrate both psychological and physical responses (43,44). The neuroanatomy of psychological stress involves higher-order brain regions, including the PC and HC, that regulate strategic decision making and memory (45,46). Conversely, the physical response to stress primarily depends on the HT via neuroendocrine mechanisms (43,47). Our data suggest that transcriptional rhythms in the brain of mice exposed to social defeat lose oscillations in the HC while gaining rhythms in the PC. Whether these variations in rhythmicity have substantive impacts on cognitive functions remains to be demonstrated. Notably, when comparing stress-resilient and stress-susceptible mice, we did not observe that the rhythmic transcriptome in these higher brain regions differed between the groups as much as the HT, suggesting that the physical response to stress is potentially a better target in the search for strategies to prevent or treat stress-induced depression. This notion is substantiated by the profound distinction in the circadian transcriptional response in the liver and HT of resilient mice. Notably, these changes were not associated with an altered expression of the hepatic molecular clock. In fact, this is consistent with previous investigations that have demonstrated that the liver clock is not influenced by chronic stress, while the clocks in the lung and kidney are disrupted (48). The effects on the molecular clock in the lung and kidney have been shown to be mediated via the hypothalamic-pituitary-adrenal axis (49). In fact, our data show loss of rhythmicity in serum corticosterone levels of susceptible mice, which is most likely a major contributor to the systemic differences observed between the groups. Furthermore, the processes at play may extend beyond neuroendocrine processes such as the hypothalamic-pituitary-adrenal axis and could also involve metabolic signaling (50). The mechanisms involved likely comprise several layers of complexity.

Metabolites serve as crucial signaling molecules and exhibit temporal coordination across different organs including the liver and HT (34,35). Our data show that social defeat had a significant impact on systemic circadian metabolism, with changes in sphingomyelin and bile acid metabolism that were specific to resilient mice. Sphingomyelins play a role in synaptic plasticity, suggesting their potential involvement in mediating the transcriptional rhythms in the HT of resilient mice (51). Furthermore, bile acids possess neuroprotective properties (38) and could potentially serve as liver-brain signaling molecules essential for coping with social defeat-induced stress. In fact, taurodeoxycholate, one of the secondary bile acids that lost rhythmicity in susceptible mice, has been shown to have antidepressant actions when given to mice (52).

### Limitations of the Study

One limitation of the current study is that only male mice were used. The role of the ERα (estrogen receptor α) pathway in enhancing stress resilience is identical in males and females, although the downstream transcriptional mechanisms of ERα action exhibit sex specificity (53). Additionally, the experimental protocols have some limitations that might have affected the data. We measured the body weight and food intake of a subset, but not all, of the mice, although they were distributed across all groups. Our circadian tissue harvest commenced 24 hours after the SIT, which resulted in a potential time discrepancy of up to 20 hours between mice sacrificed over the circadian cycle. Most critically, all data and conclusions in this article are correlational in nature. Future studies need to address the causal relationship between the transcriptional rhythms in brain regions and peripheral tissues and stress adaptation.

### Conclusions

In conclusion, our dataset provides a comprehensive overview of the circadian transcriptional and metabolomic landscape in the context of social stress, revealing that circadian signatures in gene expression and metabolism characterize stress-resilient and stress-susceptible behavioral phenotypes. Notably, we identified hypothalamic-hepatic coordination, along with circulatory metabolism, as promising focal points for addressing stress-induced depressive-like behaviors. This work has the potential to inspire future investigations of chronotherapies that target depression and other mental disorders through strategic metabolic interventions.

## Supplementary Material

Supplementary Table

Key Resources Table
