# Supplementary material for: Resilience to Chronic Stress Is Characterized by Circadian Brain-Liver Coordination
Source: Biol Psychiatry Glob Open Sci. 2024 Aug 23;4(6):100385. doi: 10.1016/j.bpsgos.2024.100385 (PMC11462208; doi:10.1016/j.bpsgos.2024.100385)

SUPPLEMENTARY INFORMATION

Resilience to Chronic Stress Is Characterized by Circadian Brain-Liver Coordination

Savva *et al.*

SUPPLEMENTAL FIGURES

Figure S1

Functional enrichment analysis of rhythmic genes generated by *DryR* in control (model 2) mice. Cnet plots represent rhythmic genes that are enriched in GO terms or KEGG pathways with a cut-off of p-adjusted value <0.05. The size of the bubble represents the size of the enriched pathway. Different line colors represent the different GO terms or KEGG pathways. N = 3 mice per time-point and group.

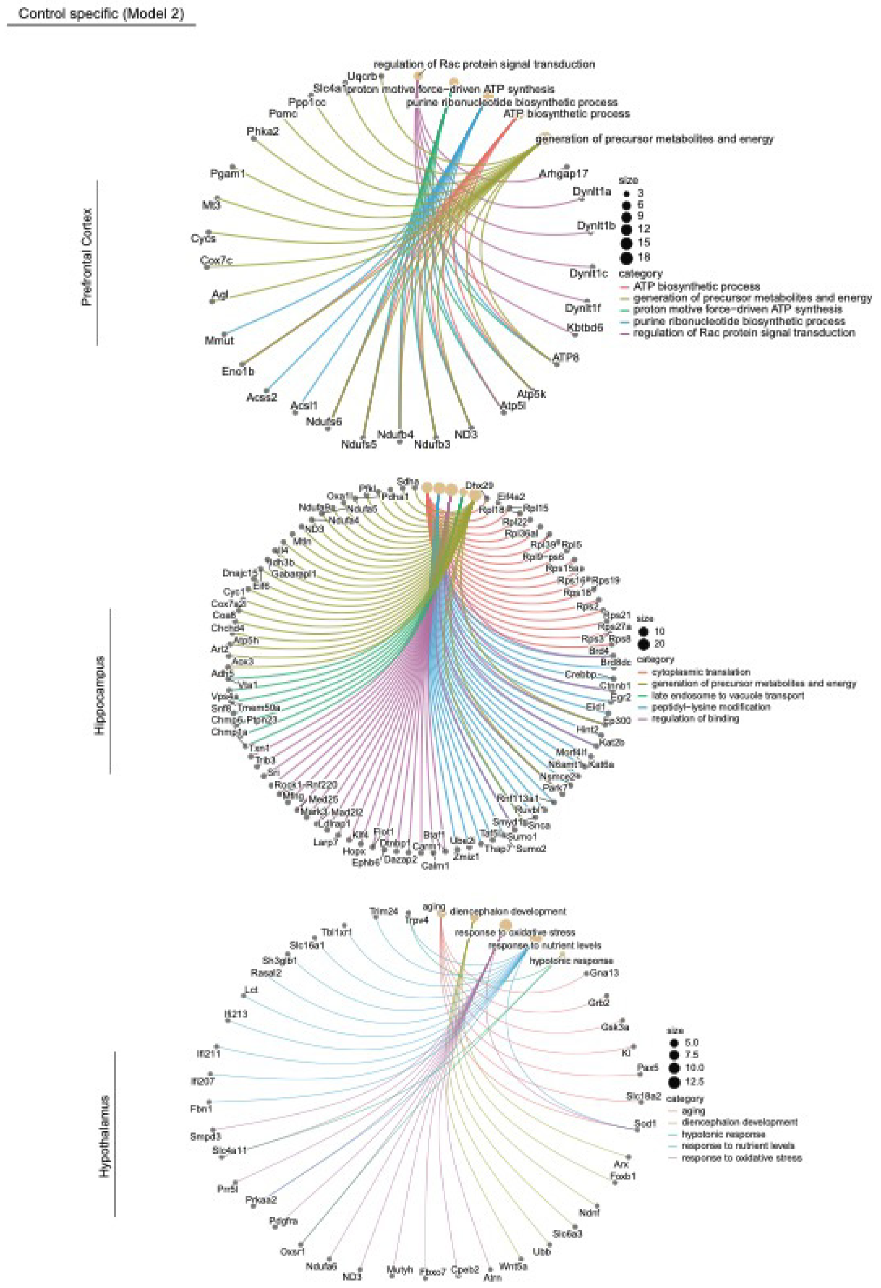

Figure S2

Functional enrichment analysis of rhythmic genes generated by *DryR* in resilient (model 3) mice. Cnet plots represent rhythmic genes that are enriched in GO terms or KEGG pathways with a cut-off of p-adjusted value <0.05. The size of the bubble represents the size of the enriched pathway. Different line colors represent the different GO terms or KEGG pathways. N = 3 mice per time-point and group.

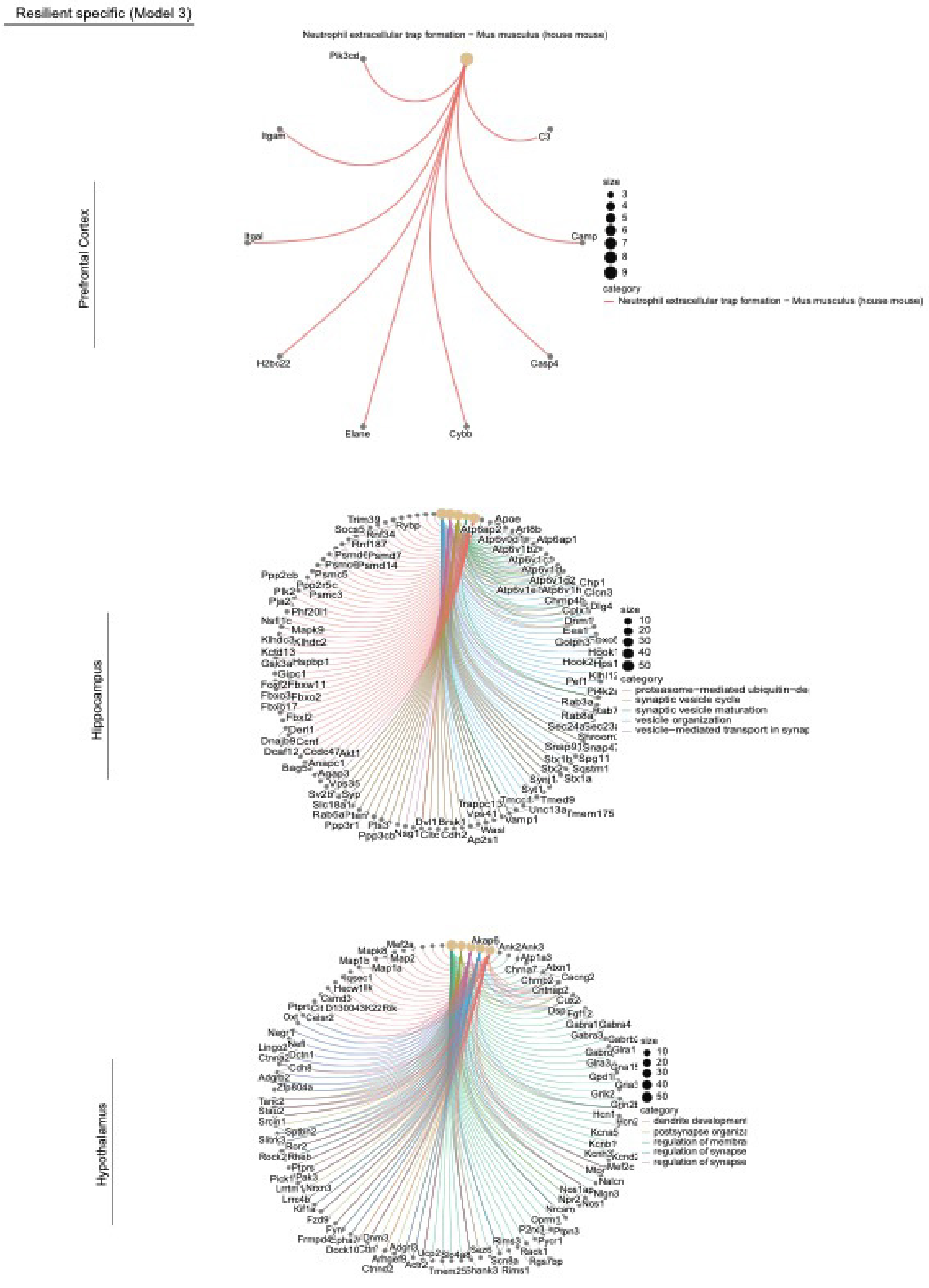

Figure S3

Functional enrichment analysis of rhythmic genes generated by *DryR* in control and resilient (model 5) mice. Cnet plots represent rhythmic genes that are enriched in GO terms or KEGG pathways with a cut-off of p-adjusted value <0.05. The size of the bubble represents the size of the enriched pathway. Different line colors represent the different GO terms or KEGG pathways. N = 3 mice per time-point and group.

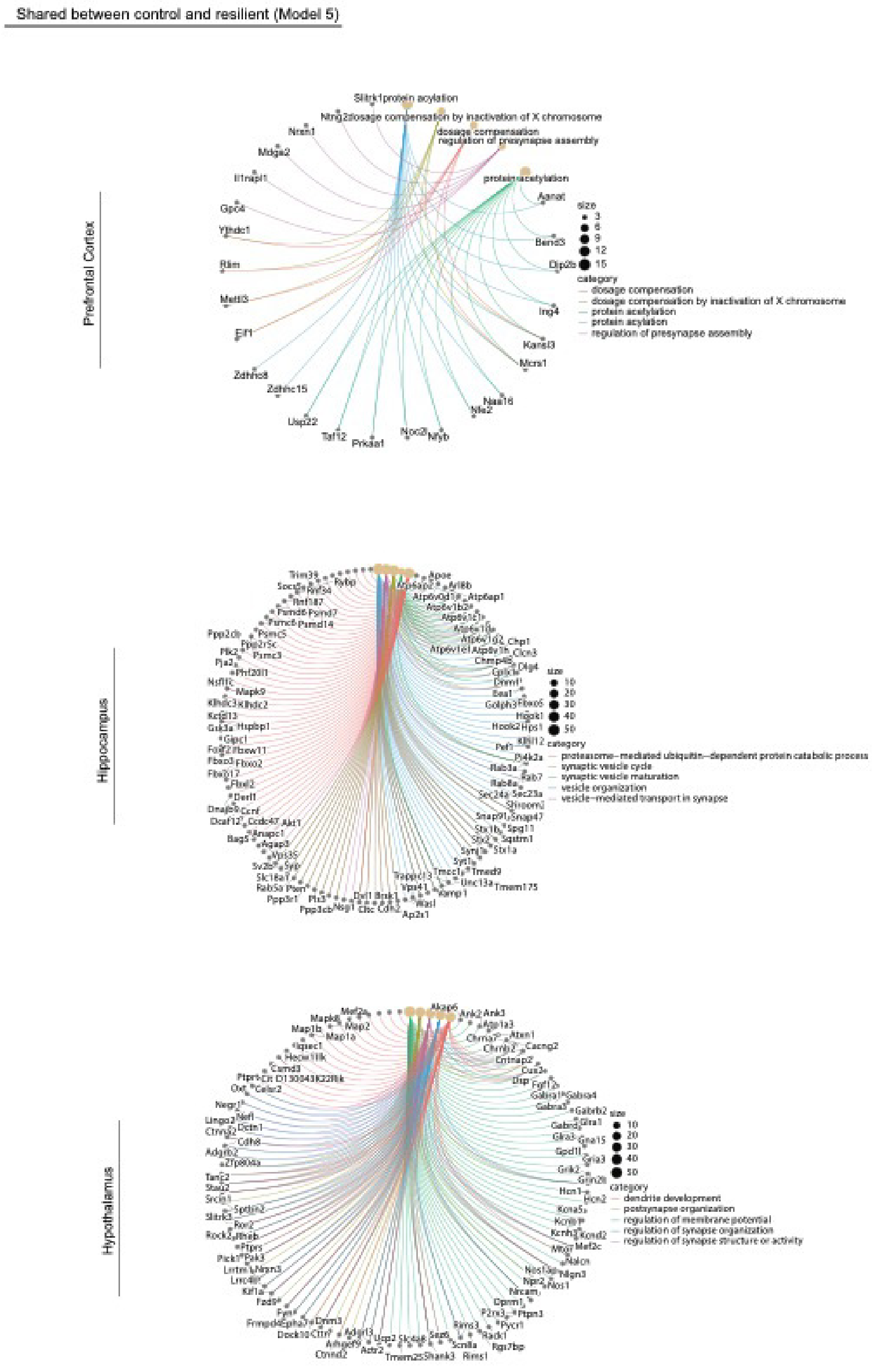

Figure S4

Functional enrichment analysis of rhythmic genes generated by *DryR* in resilient and susceptible (model 9) mice. Cnet plots represent rhythmic genes that are enriched in GO terms or KEGG pathways with a cut-off of p-adjusted value <0.05. The size of the bubble represents the size of the enriched pathway. Different line colors represent the different GO terms or KEGG pathways. N = 3 mice per time-point and group.

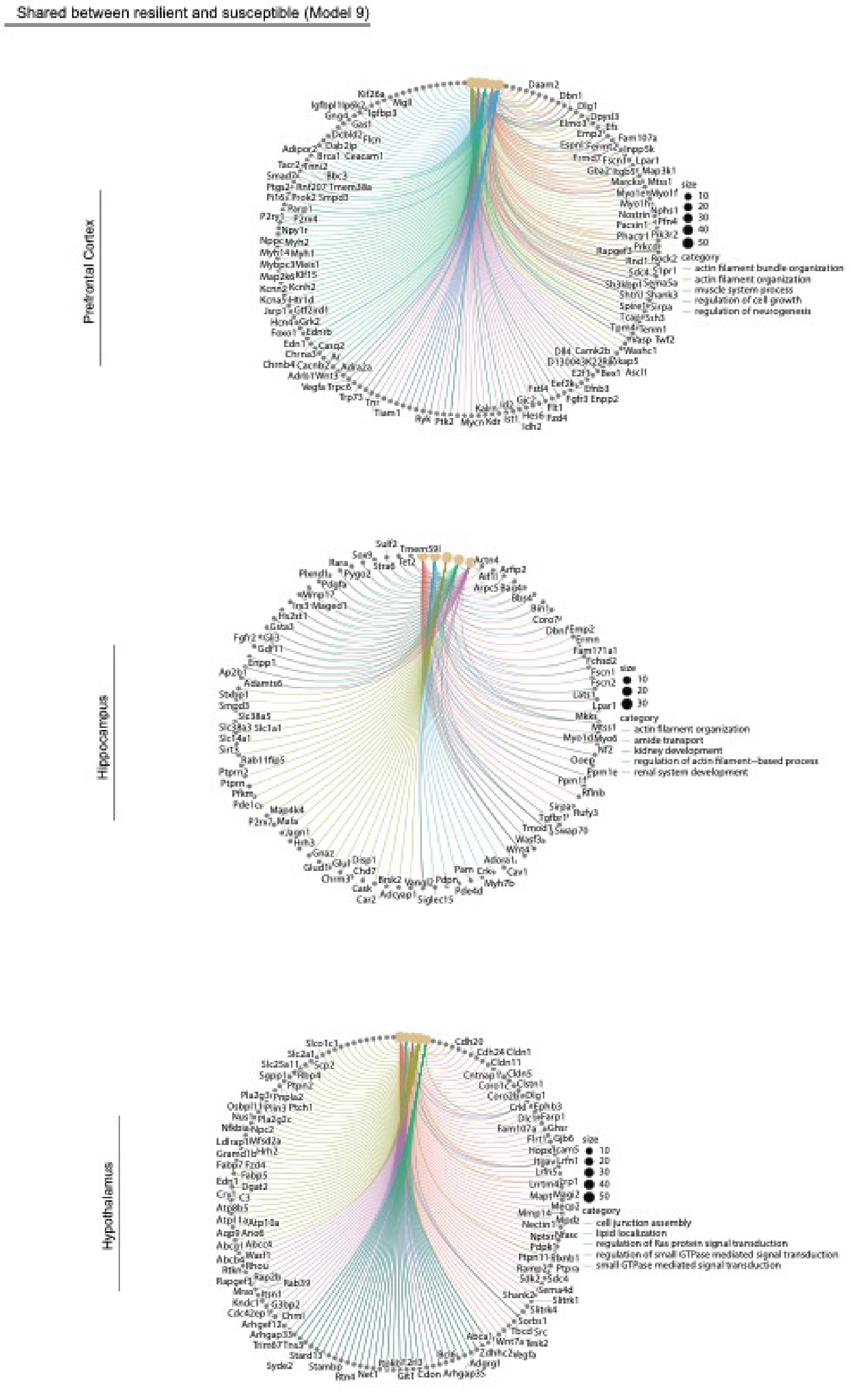

Supplement: Supplementary Figures S1-S4 [file mmc3.pdf]
